# Milk glucosidase activity enables suckled pup starch digestion

**DOI:** 10.1186/s40348-016-0032-z

**Published:** 2016-02-01

**Authors:** B. L. Nichols, M. Diaz-Sotomayor, S. E. Avery, S. K. Chacko, D. L. Hadsell, S. S. Baker, B. R. Hamaker, L. K. Yan, H. M. Lin, R. Quezada-Calvillo

**Affiliations:** Children’s Nutrition Research Center, Baylor College of Medicine and Texas Children’s Hospital, Houston, TX USA; Ciep-facultad de Ciencias Quimicas, Universidad Autonoma de San Luis Potosi’, San Luis Potosi’, Mexico; Whistler Center for Carbohydrate Research, Purdue University, West Lafayette, IN USA; University of Idaho, Moscow, ID USA; Department of Pediatrics, State University of New York, Buffalo, NY USA

**Keywords:** Weaning, Suckling, Starch digestion, Maltase-glucoamylase, Sucrase-isomaltase, Glucogenesis from ^13^C-starch

## Abstract

**ᅟ:**

Starch requires six enzymes for digestion to free glucose: two amylases (salivary and pancreatic) and four mucosal maltase activities; sucrase-isomaltase and maltase-glucoamylase. All are deficient in suckling rodents.

**Objective:**

The objective of this study is to test ^13^C-starch digestion before weaning by measuring enrichment of blood ^13^C-glucose in maltase-glucoamylase-null and wild-type mice.

**Methods:**

Maltase-glucoamylase gene was ablated at the N-terminal. Dams were fed low ^13^C-diet and litters kept on low ^13^C-diet. Pups were weaned at 21 days. Digestion was tested at 13 and 25 days by intragastric feeding of amylase predigested ^13^C-α-limit dextrins. Blood ^13^C-glucose enrichment was measured by gas chromatography combustion isotope ratio mass spectrometry (GCRMS) using penta-acetate derivatives.

**Results:**

Four hours after feeding, blood ^13^C-glucose was enriched by 26 × 10^3^ in null and 18 × 10^3^ in wild-type mice at 13 days and 0.3 × 10^3^ and 0.2 × 103 at 25 days (vs. fasting *p* = 0.045 and *p* = 0.045). By jejunal enzyme assay, immunohistochemistry, or Western blots, there was no maltase activity or brush border staining with maltase-glucoamylase antibodies at 13 days, but these were fully developed in the wild-type mice by 25 days. In 13-day null mice, luminal contents were stained by maltase-glucoamylase antibodies. Lactating the mammary gland revealed maltase-glucoamylase antibody staining of alveolar cells. Reverse transcription/polymerase chain reaction (RT/PCR) of lactating glands revealed a secreted form of maltase-glucoamylase.

**Conclusions:**

(1) ^13^C-α-limit dextrins were rapidly digested to ^13^C-glucose in 13-day mice independent of maltase-glucoamylase genotype or mucosal maltase activity. (2) This experiment demonstrates that a soluble maltase activity is secreted in mouse mother’s milk which enables suckling pup starch digestion well before brush border enzyme development. (3) This experiment with ^13^C-α-limit dextrins needs to be repeated in human breast fed infants.

The word “mammal” is from the scientific name *Mammalia* derived from the Latin *mamma* (“teat”). All female mammals suckle their young with milk which is secreted from apocrine mammary glands. Only the young are fed mother’s milk, and the process of suckling cessation is called *weaning*. For human infants, supplementation of maternal nursing is called complementary feeding or beikost [1]. The German word *beikost* translates as “infant foods other than milk or formula.” Starchy foods are the first offered as beikost in most cultures [2]. The introduction of beikost begins the weaning process and introduces environmental vectors stressing the maternal-infant dyad. The presently recommended age for introduction of complementary feedings to the suckling infant is 6 months [2]. One of the arguments for this controversial recommendation offered by learned committees is the physiologic delay of amylase production and secretion in normal infants. Pediatricians worldwide know that many mothers start offering beikost in the first months of life and that this common practice is usually rewarded by infant behavior. On the other hand, it is believed that in developing counties, much of the stunting of infant growth is due to poor nutritional quality and environmental contamination of complementary feedings [3]. With a general hypothesis that the starch offered in complementary foods is nutritionally available to in the suckling infant, we designed the following set of experiments in a mouse model. As in the human infant, developmental amylase insufficiency is present in the mouse. In suckling rodents, amylase and mucosal glucoamylase insufficiency are well documented [4]. This led to our experimental hypothesis that the suckling mouse can digest starch before the age of formal weaning, 21 days. The maltase-glucoamylase (Mgam) gene was ablated at the N-terminal expressing the membrane binding domain [5], and this hypothesis was tested in mucosal Mgam knockout (KO) and wild-type (WT) mice before and after weaning. In these experiments, it was shown that suckling mice begin to consume the adult diet by 15 days and that they can digest food starch before that age. The source of the starch digesting glucosidase activity was shown to be maternal milk which is expressed from the lactating alveolar cells of the mammary gland in a smaller molecular weight soluble form when compared to the membrane-bound form of the wild-type adult mouse jejunum.

## Results and discussion

### Mgam KO on in vitro suckling starch digestion

**Fig. 1:**
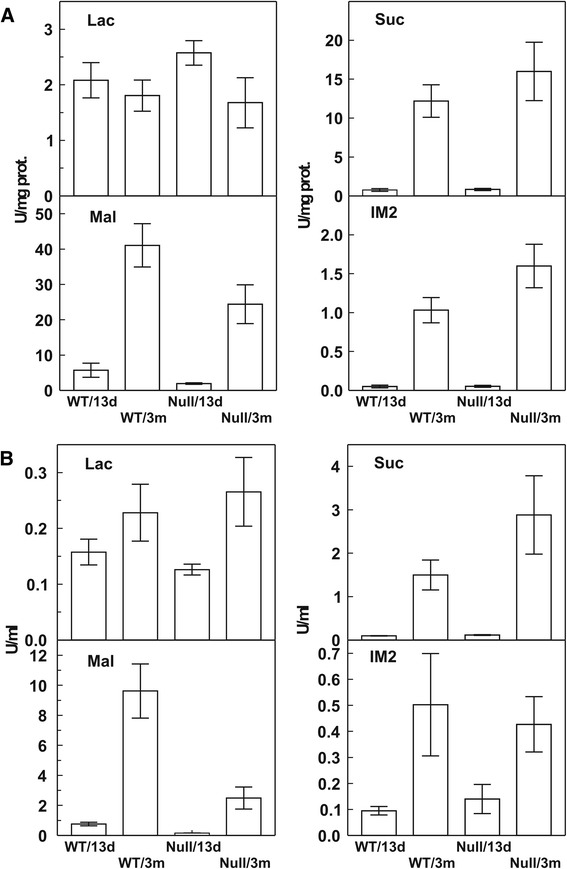
Dahlqvist disaccharidase assays from the mid-jejunum of suckling and weaned mice with Mgam KO or WT genotypes (*n* = 8). Weaning was by separation from the litter at 21 days. Tissues were collected at 13 days (suckling) and 90 days (weaned). The lumen was first flushed with EDTA PBS buffer, and then, tissue was collected and homogenized in PBS buffer and analyzed as unit per milligram protein (**a**). The luminal flush (**b**) was assayed for activity per milliliter of collected fluid. Note that the homogenate activities for maltose, sucrose, and isomaltose substrates increased after weaning in the WT but not in the null mice. Note also the difference in ordinate scales between (**a**) and (**b**)

Methods: Genotyped mice were bred to produce Mgam KO and WT pups [5, 6]. Dates of birth were closely monitored for accuracy of ages. All the suckling mice had clotted milk visible in stomach at time of sacrifice. One-centimeter segments were removed from the mid-jejunum and conserved at −70 °C for subsequent glucosidase assays [6]. After flushing the lumen, the tissue was homogenized in phosphate-buffered saline (PBS) buffer with ethylene diamine tetra-acetic acid (EDTA). Aliquots of the homogenate and flush were assayed for glucose production from maltose substrate by the Dahlqvist glucose-oxidase method. Results: After weaning at 21 days, there was a clear difference between null and WT genotype (Fig. 1) jejunum homogenate α-glucosidase activities at 13 and 90 days of age in eight null and eight WT mice. Conclusion: There was confirmation that mucosal-bound Mgam was ablated in weaned KO mice and that all α-glucosidase activities were absent in both null and WT suckling mice.

### In vivo digestion of starch in suckling mice

Methods: To test starch digestion, we used amylase-pretreated UL ^13^C-starch limit dextrin (^13^C-LDx) [7] which was fed by gavage to 13-day-old suckling mice with either KO or WT Mgam genotypes. These same genotypes were tested again at 25 days, 4 days after weaning. Mating of four to six females fed low ^13^C-diet (TD.06089, based upon rice starch) for each mouse genotype, enough to produce four litters of the same (±6 h) age for each. The resulting litters were kept under low ^13^C-feeding (lactating) and housing [8]. Twenty-four mice were required for the study. At 21 days, the mice were weaned in individual cages to low ^13^C-diet with feeding permitted 24 h. Four mice were taken from the null and four from the WT genotypes, and blood and tissue were collected 4 h after intragastric feeding of ^13^C-LDx. Trunk blood was collected in fluoride/EDTA tubes at 4 °C and spun and frozen at 70 °C until analysis. Enrichments of plasma ^13^C-glucose (starch glucogenesis) were measured by gas chromatography combustion isotope ratio mass spectrometry (GCRMS) using the penta-acetate derivative. Jejunal α-glucosidase activities were measured as above. Results: The mucosal α-glucosidase phenotypes confirmed the genotypes of the weaned mice. Four hours after ^13^C-LDx feeding, blood ^13^C-glucose was enriched by 26 × 10^3^ in null and 18 × 10^3^ in WT at 13 days and 0.3 × 10^3^ and 0.2 × 103 at 25 days (vs. fasting *p* = 0.045 and *p* = 0.045). Conclusion: Fed ^13^C-LDx was rapidly digested and transported to enrich blood ^13^C-glucose in 13- and 25-day mice independent of Mgam genotype or jejunal homogenate phenotype.

### C-terminal Mgam epitope in suckling jejunum lumen

**Fig. 2:**
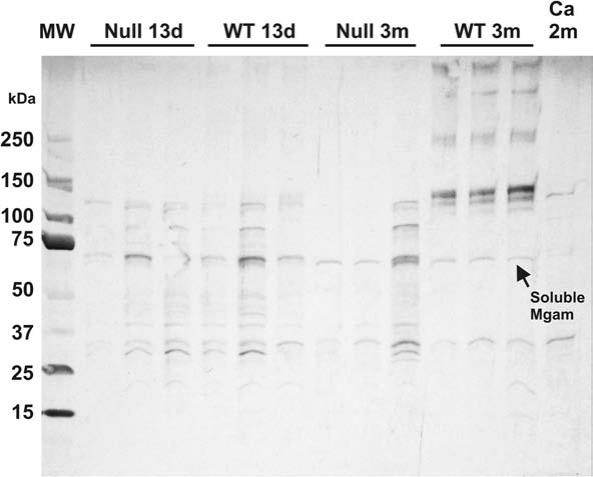
Western blots of mid-jejunal homogenates from mice with Mgam KO or WT genotypes before (13 days) and after (90 days) weaning. Mgam KO and WT were analyzed at each age (*n* = 3). Molecular weight standards are run in the left two columns and a negative control, tissue from Mgam deficient adult CBA Ca strain mouse, in the right column. The rabbit anti-mouse Mgam polyclonal served as primary antibody (see text). Note that only the 90-day WT tissue had the characteristic bands associated with Mgam but that smaller bands were recognized in all mice. These smaller bands were also visualized on Western blots of mouse milk (see text)

Methods: Recombinant mouse C-terminal Mgam was used to immunize rabbits [9]. The resulting polyclonal antibody (pAb) was specific as evidenced by blocking with the immunizing peptide and absence of reaction with C-terminal sucrase-isomaltase (Si) immunoprecipitates from the mouse mucosa. Results: Western blots of whole jejunal homogenates of triplicate null and WT 13-day suckling mice revealed three bands at 60–120 KDa that were also visualized at 3 months, but the mature pattern of five bands at 130–400 was only seen in the weaned WT homogenates (Fig. 2). Immunohistology of jejunum revealed staining of luminal milk in both null and WT 13-day mice but only staining of the brush border in the 3-month WT jejunum. Conclusion: A specific rabbit antibody against mouse Mgam recognizes a smaller MW epitope in suckling mice which is visualized as milk residue in the lumen of the jejunum. The higher MW Western blot bands and jejunal brush border staining patterns are only visualized in the weaned WT mice.

### C-terminal Mgam epitope in mouse milk and lactating gland

Method: Mammary tissue was removed from a WT lactating mouse at 20 days post-partum. Milk was obtained by vacuum milking from three 20-day post-partum lactating dams and frozen for Western blotting. By immunohistology, the lactating gland was diffusely stained with the Mgam pAb at low power, and staining of the milk-producing alveolar cells and ductal lumen contents was clearly visualized on higher power. The Western blot from three 20-day lactating C57BL/6J mouse milks revealed the expected higher MW Mgam bands from control WT mouse jejunum and only the lower MW bands similar to those of suckling mice in Fig. 2. Conclusion: The epitope for glucoamylase is visualized by immunohistology of alveolar cells of the mouse lactating mammary gland and is also recognized by Western blots of mouse milk.

### Secreted form of Mgam mRNA in lactating gland

Method: Lactating mammary glands were harvested from ten different strains of mice (129S1/SvImJ, C57BL/6J, CBA/J, BTBRT<+>tf/J, Qsi5, MA/MyJ. A/J, Sea/GnJ, FVB/NJ, and CZECHIII/Eij strains) at 20 days post-partum. The tissues were snap frozen and stored for assay. Method and results: WT mouse jejunal RNA served as positive control. RNA was extracted as previously described [10]. A first reverse transcription/polymerase chain reaction (RT/PCR) had 5′ end primers anchored in exon 1 for both Si (exon 1 to exon 6) and Mgam (exon 1 to exon 7), and only mouse jejunal RNA samples were amplified on the first RT/PCR. In a second RT/PCR, Si (exon 16 to exon 18) did not amplify from the mammary gland RNAs, but the N-terminal Mgam (exon 3 to exon 7) did amplify which suggests that exon 1 for Mgam is spliced out for lactating mammary gland [10]. Conclusion: RT/PCR of lactating glands from ten different mouse strains revealed a message for a secreted form of Mgam, lacking the membrane binding coding exon.

### Weaning process in suckling mice

From an animal management perspective, mouse weaning took place at 21 days of life when the pups are separated into individual cages. From a nutritional perspective, the feeding on mouse chow was begun during suckling. In the Mgam-null mouse, the coexistence of stained milk globules and unstained undigested plant structures were evident by age 15 days. This is consistent with the term *weaning* which implies a gradual transition from maternal to external food sources.

### Weaning

From a chemical perspective, there is a major shift in food carbohydrate substrate affinity during weaning. Lactose is the major disaccharide in mother’s milk. It is linked by a β-linkage between glucose and galactose. The digestible dietary carbohydrates of foods are, with the exception of milk, all α-linked glucose oligomers, while the indigestible cell structures, usually distally fermented, are β-linked fibers. In this context, mammalian weaning is a developmental shift of small intestinal β- to α-glucosidic substrate digestion, a shift from a carnivorous to an omnivorous diet in human infants.

The major limitation to this investigation was the necessary use of a mouse Mgam KO model [6]. In contrast to the suckling mouse, humans have full mucosal Mgam and Si activities in jejunal mucosa from before birth [11]. The physiologic delay in salivary and pancreatic amylase production and secretion [12, 13] parallels the rodent [14] and other model species. In our in vivo experimental model in mice, we removed the question about amylase maturation from consideration by exhaustive pre-digestion the test starch with amylase to make limit dextrins (LDx) [8]. Mucosal activities were tested in vitro with maltose made by amylase digestion of starch [7]. Mucosal glucogenesis from LDx is commonly termed α-glucosidase activity although the same mucosal activity is also recognized with maltose substrate, the major product of starch hydrolysis by amylase. As assayed in vitro, mucosal activity is a composite of four different enzymes expressed from two genes, maltase-glucoamylase (Mgam) and sucrase-isomaltase (Si), each with an N-terminal and C-terminal maltase activity. There are differences in rates and substrate specificities of the four maltase activities [7]. The C-terminal of Mgam is the most active for maltose and long chain or branched oligosaccharide substrate glucogenesis. Mgam activities are almost independent of amylase hydrolysis, but Si activities are greatly amplified by amylase pre-hydrolysis of starches. This has led to the view that glucoamylase activities suffice for starch digestion during physiologic amylase insufficiency [15]. This set of experiments suggests that secreted enzymes in mother’s milk participate in digestion of complementary starch in mice. Reported studies of human milk demonstrate the presence of amylase peptide and activity as part of the functional components in human milk [16–19]. Could the amylase activity secreted in mother’s milk amplify the glucogenic activities present in the human infant’s small intestine?

### Pediatric nutritional implications

There is no direct proof that human infants can digest complementary food starches in the small intestine, but indirect evidence exists. Studies of starch feeding show that both whole cereal starch and various maltodextrins (MDx) are digested by the young infant [20, 21]. Evidence from complementary feeding studies of nursing infants in affluent settings reveals that complementary foods amplify energy intakes and sustain growth [22]. However, in challenging environments, complementary feeding may fail to sustain infant growth [3]. The problem of infant stunting is of global significance [3, 23, 24]. While not addressing this global pediatric issue directly, this experiment shows that suckling starch digestion is enabled by mouse maternal nursing and suggests that human breast milk feeding may enable amplified starch digestion in the suckling infant. This hypothesis is testable by the minimally invasive ^13^C-LDx feeding procedure used in experiment 2 of this mouse investigation.

## Abbreviations

EDTA: ethylene diamine tetra-acetic acid
GCRMS: gas chromatography combustion isotope ratio mass spectrometry
KO: knockout
LDx: α-limit dextrins
MDx: maltodextrins
Mgam: maltase-glucoamylase
MW: molecular weight
pAb: polyclonal antibody
PBS: phosphate-buffered saline
RT/PCR: reverse transcription/polymerase chain reaction
Si: sucrase-isomaltase
UL: universally ^13^C-labeled
WT: wild type
